# A Fiber Optic PD Sensor Using a Balanced Sagnac Interferometer and an EDFA-Based DOP Tunable Fiber Ring Laser

**DOI:** 10.3390/s140508398

**Published:** 2014-05-12

**Authors:** Lutang Wang, Nian Fang, Chunxu Wu, Haijuan Qin, Zhaoming Huang

**Affiliations:** Key Laboratory of Specialty Fiber Optics and Optical Access Networks, School of Communication and Information Engineering, Shanghai University, No.149, Yanchang Road, Shanghai 200072, China; E-Mails: nfang@staff.shu.edu.cn (N.F.); wuchunxu-1580@163.com (C.W.); shuqhj@163.com (H.Q.); zmhuang@mail.shu.edu.cn (Z.H.)

**Keywords:** fiber optic sensors, acoustic sensing, Sagnac interferometer, polarization, DOP, partial discharges

## Abstract

A novel fiber-optic acoustic sensor using an erbium-doped fiber amplifier (EDFA)-based fiber ring laser and a balanced Sagnac interferometer for acoustic sensing of the partial discharge (PD) in power transformers is proposed and demonstrated. As a technical background, an experimental investigation on how the variations of the fiber birefringence affect the sensor performances was carried out, and the results are discussed. The operation principles are described, and the relevant formulas are derived. The analytical results show that an EDFA-based fiber ring laser operating in chaotic mode can provide a degree of polarization (DOP) tunable light beam for effectively suppressing polarization fading noises. The balanced Sagnac interferometer can eliminate command intensity noises and enhance the signal-to-noise ratio (SNR). Furthermore, it inherently operates at the quadrature point of the response curve without any active stabilizations. Several experiments are conducted for evaluating the performances of the sensor system, as well as for investigating the ability of the detection of high-frequency acoustic emission signals. The experimental results demonstrate that the DOP of the laser beam can be continuously tuned from 0.2% to 100%, and the power fluctuation in the whole DOP tuning range is less than 0.05 dBm. A high-frequency response up to 300 kHz is reached, and the high sensing sensitivity for detections of weak corona discharges, as well as partial discharges also is verified.

## 1. Introduction

Partial discharges are small electrical sparks that occur when voids exist within or on the surface of the high-voltage dielectric material inside power appliances, such as power transformers. As the insulation degrades, the number and magnitude of the PD pulse will increase rapidly, and the PD signal level becomes higher than before. Therefore, the PD is becoming one of the major sources and symptoms of the deterioration of the dielectric material for people to judge the deterioration of insulation and estimate the remaining life of a power transformer [1].

As commonly known, PD pulses emit weak ultrasonic wave signals typically ranging from 20 kHz to 300 kHz. Although the traditional piezoelectric transducer (PZT)-type of acoustic emission (AE) sensor can provide high sensitivity in sensing these weak ultrasonic wave signals, the huge electromagnetic stresses around power transformers often limit it from being used widely, especially inside power transformers. On the other hand, as an alternative, fiber-optic interferometer-based acoustic sensors with the property of immunity from electromagnetic interferences [2,3] can be employed within an environment, such as inside or outside power transformers, for PD monitoring, as well as source locating [4–10]. In fiber-optic acoustic sensors, there are several major types that have been developed recently, which include diaphragm-based Fabry–Perot interferometric sensors [11,12], fiber Bragg grating (FBG)-based sensors [13,14] and Sagnac interferometric sensors [15–17], as well as fiber laser-based interferometric sensors [18]. These types of sensors, depending on their structures, have different sensing performances, in terms of the sensitivity and frequency response, and have different applicabilities, such as being capable of being placed inside or outside transformers or both. For most types of sensors, however, the working state often suffers from the effects of variations in environmental temperature and needs to be stabilized all the time with sophisticated control methods or specially designed compensatory structures [12]. As a counter case, the Sagnac interferometric sensor shows its superior stability against environmental temperature influences, due to its truly path-matched interference mode. Beside this, the Sagnac interferometric sensor also has high sensitivity and excellent high-frequency response to ultrasonic wave signals, which render it suitable, particularly for PD acoustic sensing [5,16,19].

Like other types of fiber-optic interferometers, however, the fiber Sagnac interferometer sensor also inevitably is subject to the effect of polarization fluctuations, which are known as polarization-induced signal fading or simply as polarization fading [20–22]. This arises from the random changes of states of polarization (SOP) of two interference beams when propagating in long fiber with birefringence in the opposite direction [23]. During the fabrication of a fiber sensor coil, the ordinary single-mode fiber is stretched, bent and twisted, which creates birefringence randomly distributed along the fiber [23,24]. The fiber birefringence results in a complicated evolution of the SOP of the beam when propagating through the fiber [24]. When the external environment changes, the fiber birefringence will be modified, which, in turn, disturbs the SOPs of propagating beams, finally altering the intensity of the interference signal.

Although this problem can be resolved well by using polarization-maintaining (PM) fibers [24,25], the high cost of PM fiber itself will limit it from being used extensively. Another solution widely adopted today is using broadband, depolarized light in the Sagnac interferometer combined with a Lyot-type depolarizer [26,27]. This depolarizer is embedded in the fiber loop to randomize the SOPs of propagating beams corresponding to the optical wavelength. Thus, there is an averaging effect on birefringence-induced polarization fading [28]. Although this method can effectively minimize the birefringence effects, the sensor sensitivity, as well as the SNR, as a penalty, will obviously decrease. The reason for this is that the power of input light randomly spreads on the overall possible SOP and only a small portion with similar SOP, for example, with a deviation of the polarization angle within ±10 degrees, can generate more credible interference outputs, while the majority with large-angle deviations will interfere with each other to form many low-level interference outputs, which actually act as the background noise to deteriorate the sensor SNR. This is why in an optical interferometric sensor with a depolarizer, the actual detection outputs seem more noisy than when using a polarized light source.

According to our experimental observations, the SOP of the signal beam varies only within a limited area on the Poincaré sphere, while the center of this area is relatively stable. This implies that instead of using completely unpolarized light in the real operational environment for PD sensing, using partially polarized light may be more effective in both suppressing polarization fading and enhancing the sensor sensitivity, compared with using unpolarized light. A convenient method for measuring the relative proportion of polarization to depolarized light is to measure the DOP of the beam, as a measure of the random distribution of SOP [29], which changes from zero (completely unpolarized) to 100% (completely polarized). In this sense, the light source used in a fiber Sagnac interferometer for PD sensing should have a suitable DOP value for effectively suppressing the polarization fading without excessively reducing the sensor sensitivity.

Fiber ring lasers based on the EDFA or the semiconductor optical amplifier (SOA) can provide a variety of SOPs through fiber nonlinearity effects. The light beam from the EDFA-based or SOA-based fiber ring laser has a chaos property in both intensity and polarization states, which can be employed not only as a chaotic carrier for secure fiber optic telecommunications [30,31], but also as a depolarized light source for fiber optic sensing [32]. The fiber ring laser working in chaos mode, sometimes referred to as the chaotic fiber ring laser, can generate very rapid polarization and intensity fluctuations [29,33]. The chaotic light beam's frequency is the wide band, and the fluctuations in its time waveform are high (over the gigahertz level) [31], which means that the average of signal beams over all SOPs is very fast without obvious fluctuations in intensity. The net polarization dynamics of a chaotic fiber ring laser also have been investigated by Gregory *et al.* with a high-speed fiber optic polarization analyzer [29]. In their experiments, the Stokes parameters of the chaotic beam from an EDFA-based fiber ring laser were detected and analyzed. From their investigation results, it is obvious that the distribution state of SOP or the polarization fluctuation magnitude of the chaos beam is controllable. This feature is particularly useful for us to develop a unique chaotic laser source with a DOP tunable function for fiber-optic PD sensing.

In this article, we propose a novel fiber-optic acoustic sensor using an EDFA-based, chaotic fiber ring laser and a balanced Sagnac sensor for PD monitoring of power transformers. The chaotic fiber ring laser can provide a DOP tunable light beam with stable output power. The balanced Sagnac interferometer with the property of immunity for command intensity noises can inherently work at its quadrature point without any active stabilizations. We present experimental results for the investigation of the polarization fading induced by fiber birefringence fluctuations in Section 2 and describe the operation principles in Section 3. In Section 4, we first illustrate the experimental results of the evaluation of sensor performances and then demonstrate the results of the detection of PD signals produced through the high voltage (HV) discharges in an oil tank in our laboratory environment. Section 5 provides discussions of the advantages and features of this sensor technique. Finally, conclusions are given in Section 6.

## 2. Experimental Observations

**Figure 1.**
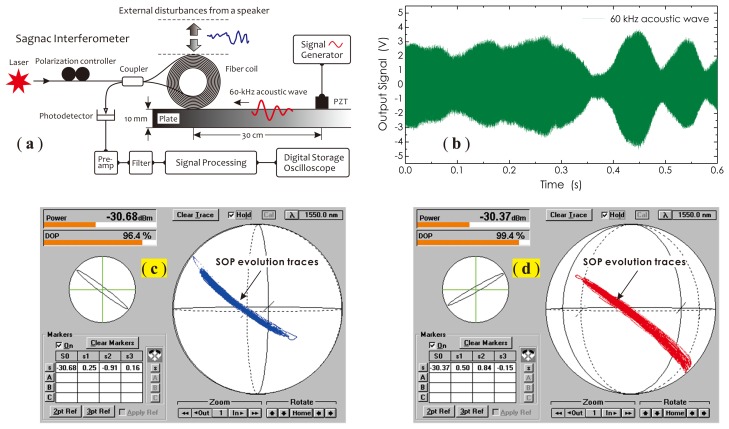
Experimental setup (**a**), the measured waveform (**b**) and SOP variations (**c**,**d**).

In the application for PD monitoring, the mechanical vibration of the power transformer itself is the biggest effect factor to disturb the fiber sensor coil, which modulates the fiber birefringence and produces the signal fading. An experimental setup shown in Figure 1a was used for observing this process. In this setup, a laser source and an ordinary Sagnac interferometer with a 2 × 2 fiber coupler were employed. A hollow fiber coil as a sensor with an inner diameter of 40 mm and a width of 50 mm was manufactured with a 1 km-long, signal-mode fiber. This fiber coil was put on an epoxy composite plate, where the silicone grease was applied prior. A PZT actuator was placed on the same side of the plate, separated by 30 cm from the fiber coil, which was driven by a 60-kHz, continuous sine wave signal. The excited ultrasonic stress wave propagating along the plate surface toward the fiber coil was detected by the Sagnac interferometer and converted into the corresponding electrical signal for waveform analyses. In this experiment, the maximum signal magnitude could be achieved by adjusting a polarization controller (PC) to change the SOP of the input beam.

In order to simulate a real operational environment, in this experiment, a speaker, driven by a signal generator, was put on the top of the coil to generate a low-frequency, large-magnitude, randomly-changed vibration. As shown in Figure 1b, significant amplitude fluctuations arising in the signal waveform could be observed, which is a typical signal waveform with polarization fading features. The reason for this is that the fiber coil vibration produced by the speaker altered the fiber stress distribution, in turn modifying the birefringence and making two counter-propagating signal beams, clockwise (CW) and counterclockwise (CCW) beams, no longer remaining consistent in their polarization states and randomly changing with time. This finally resulted in amplitude variations in the interference signal.

**Figure 2.**
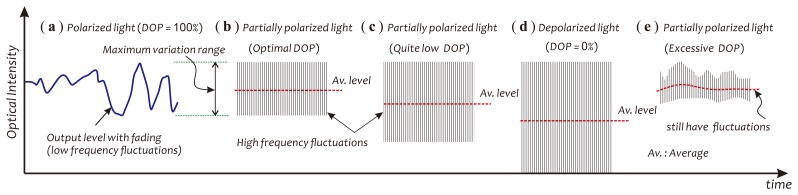
Schematics used for describing the concept of using degree of polarization (DOP) controllable, partially polarized light to relieve the polarization fading effects.

Two SOP evolution traces of signal beams under mechanical vibrations, before and after adjusting the position of the coil, were recorded with an optical polarization analyzer (Agilent/HP 8509B) and are illustrated in Figure 1c,d, respectively. Observing these two traces, one can find that the SOPs of signal beams under mechanical vibrations actually varied with time in a low-frequency manner and, however, were contained in a long and narrow zone on the Poincaré sphere. This reflects the fact that the distribution state of the fiber birefringence had been modulated by external mechanical vibrations. It also implies that for effectively relieving polarization fading effects, one can employ a DOP controllable, partially polarized light source to actively ‘disturb’ the SOPs of signal beams, so as to achieve an average effect on the polarization fading in a high-speed manner, generally up to several gigahertz. For getting a good averaging effect, the variations of the signal intensity, caused by beam SOP fluctuations in this dynamic mode, should just cover or be slightly larger than those produced by fiber birefringence fluctuations. This concept is schematically described in Figure 2.

**Figure 3.**
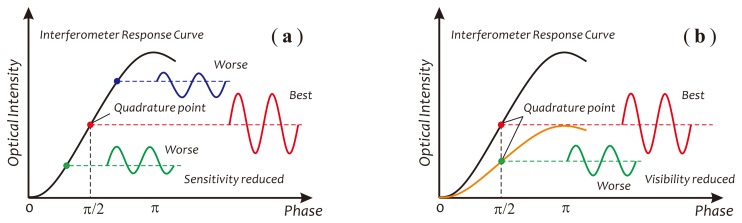
Schematics used for describing birefringence variations affecting sensor performances: (**a**) deviating from the quadrature point; (**b**) visibility reduced.

Besides the polarization fading, the fiber birefringence variations also could cause the sensor to deviate from its best working point (the quadrature point), as well as modify the fringe visibility, as depicted in Figure 3, respectively. According to previous research [20], the detection intensity of a Sagnac interferometer, under the considerations of the fiber birefringence and the polarization mode coupling in the fiber coil, can be expressed as:

(1) $$I=\frac{I_{in}}{2}[1-\cos^{2}\theta \cos\Delta \phi (t)+\sin^{2}\theta \cos(\phi_{\text{b}}+\Delta \phi (t))]$$

where *I_in_* and *I* are the optical intensity of the input and output beam, respectively. *θ* is an angle through which the SOP of the input beam is rotated by the fiber birefringence [20]. *ϕ*_b_ and Δ*ϕ*(*t*) are two phase shifts between counter-propagating beams through the fiber coil. *ϕ*_b_ is a static phase shift resulting from the fiber birefringence, usually referred to as the phase bias, while Δ*ϕ*(*t*) is another time-dependent phase shift produced by external disturbances. From Equation (1), it is clear that the working point of the sensor is dependent on the phase bias, *ϕ*_b_, and the fringe visibility is a function of *θ*. When *ϕ*_b_, as well as *θ* are equal to an odd multiple of *π*/2, the sensitivity and visibility will achieve their maximums. It is clear that any variations in the fiber birefringence will directly modify *θ* and *ϕ*_b_, thus substantially degrading sensor performances and producing erroneous outputs. The above problems have been discussed widely in much literature, and many approaches for stabilizing the working point of the sensor, as well as minimizing the undesired peripheral effects have been proposed [20–22,34,35].

## 3. Principles

**Figure 4.**
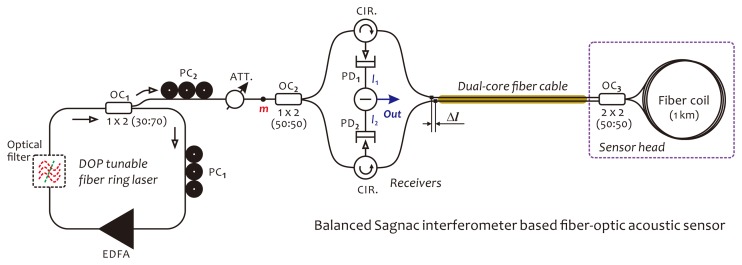
Schematic of the proposed sensor configuration. In this configuration, PC*_i_*, OC*_i_*, CIR., ATT., and PD*_i_* represent the fiber polarization controller, the fiber coupler, the optical circulator, the optical attenuator, as well as photodetector, respectively. Δ*l* is the length difference between two transmission fibers in dual-core fiber cable. EDFA, erbium-doped fiber amplifier.

**Figure 5.**
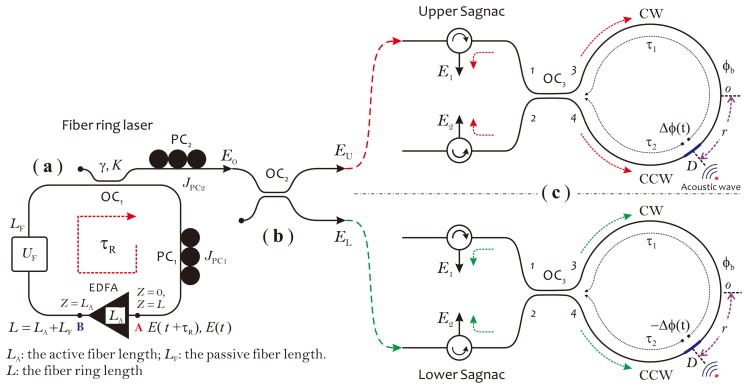
Theoretical models used to describe the principles: (**a**) for a chaotic fiber ring laser; (**b,c**) for a balanced Sagnac sensor. CW, clock-wise; CCW, counter clock-wise.

Based on our investigations, as mentioned in the previous section, in this section, we propose a novel fiber optic acoustic sensor illustrated in Figure 4 for the PD monitoring of power transformers. This sensor consists of an EDFA-based fiber ring laser as a light source and a balanced fiber Sagnac interferometer as sensor, or simply called the balanced Sagnac sensor. The fiber ring laser operating in chaotic mode can provide a partially polarized output beam with the required DOP value. The DOP of the output beam is adjustable according to different operating environments for effectively suppressing the polarization fading in detection signals. The balanced Sagnac sensor is specially designed for PD sensing in the HV environment, without using any metal parts, as well as electric-drive components in the sensor head. Furthermore, the sensor system is required for stable work at the quadrature point. Moreover, it is required to cancel out the intensity noises appearing in detection signals, which usually arise from a power fluctuation in the laser output, due to the high sensitivity of the fiber ring laser to ambient acoustic noises. The balanced Sagnac sensor actually consists of two individual, identical Sagnac interferometers, both sharing a fiber coil as the sensor head and using two relevant, low-coherent probe lights with identical optical power. The sensor head, including a fiber coil made of a 1 km-long single-mode fiber and a 2 × 2 fiber coupler, is connected to the system with a dual-core fiber cable. This fiber cable can be extended to over 1 km in length, if required in actual applications. There is a length difference, denoted as Δ*l*, that exists between two transmission fibers in the dual-core fiber cable. This length difference should be longer than the coherence length of the laser beam in order to make two probe lights incoherent. In this sensor configuration, since two detection outputs, *I*_1_ and *I*_2_, respectively, coming from the upper and lower Sagnac interferometers, are in anti-phase, the final detection output can be obtained through a differential operation achieved in the electrical domain with *I*_1_ and *I*_2_. With this operation, the command-mode intensity noises from optical power fluctuations appearing in both *I*_1_ and *I*_2_ can be canceled out completely. In this way, the performances of the sensor system in terms of sensitivity, long-term stability, as well as SNR will be greatly enhanced. In the fiber ring laser, an optical filter can be employed as an option to determine a unique lasing wavelength, which usually is required in a wavelength division multiplexing (WDM) sensor network. In the following two subsections, we use a schematic diagram, as illustrated in Figure 5, to describe the operation principle of the fiber ring laser and the balanced Sagnac sensor, respectively.

### 3.1. Principle of the Chaotic Fiber Ring Laser

According to theoretical analyses provided in [36], the chaotic dynamics of an EDFA-based fiber ring laser can be described with a set of delay differential equations. When the beam propagates through the fiber ring, the small physical effects, such as the Kerr nonlinear effect, the medium's polarization of silicon material and the group velocity dispersion (GVD), as well as the linear birefringence of the fiber, which directly involve the electric field of the beam, can accumulate up to such a high level, as to effect the laser oscillation states to generate different working modes. For an electric field, **E**(t), with two orthogonal polarization components, *E_x_*(*t*) and *E_y_*(*t*), propagating in the fiber ring, after circulating one turn, this can be well approximately as:

(2) $$E(t+\tau_{R})=\sqrt{\left(1-\gamma )(1-K\right)}\cdot R\cdot e^{j\bar{\eta }}\cdot J_{{\text{PC}}_{1}}\cdot U_{\text{F}}\cdot P\{E(t)\}$$

where *τ_R_* is the round-trip time of the beam in the fiber ring and γ and *K* represent the excess loss and the field intensity coupling coefficient of the fiber coupler, OC_1_, respectively. **R** = **diag**(*R_x_, R_y_*) denotes an absorption matrix of the fiber, *η̂* is an average propagation phase shift, **J**_PC_1__ is a Jones matrix of PC_1_ and **U**_F_ represents the birefringence matrix of total fiber. ( 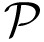) is defined as a propagation operator [36], which transports **E**(*t*) from the beginning (Point A) to the end (Point B) of the EDFA (see Figure 5a).

A polarization controller, PC_1_, in the fiber ring manages the overall birefringence of the fiber ring, so as to control the type of system dynamics and to tune the DOP of the laser beam. By adjusting PC_1_, the different DOP values can be obtained. The electric field of the output beam after passing across OC_1_ and another polarization controller, PC_2_, is expressed as $E_{0}(t)=j\sqrt{K}{\text{J}}_{{\text{PC}}_{2}}E(t)$, where **J**_PC_2__ is a Jones matrix of PC_2_. Generally, **E**_0_(*t*) is elliptically polarized and can be expressed as a Jones vector [37]:

(3) $$E_{0}(t)=\left(\begin{array}{c} E_{0x}(t)\\ E_{0y}(y) \end{array}\right)=E_{0}\left(\begin{array}{l} \cos[\beta +\Delta \beta (t)]\\ \sin[\beta +\Delta \beta (t)]e^{j(\alpha +\Delta \alpha (t))} \end{array}\right)$$

where $E_{0}=\sqrt{{\left|E_{0x}\right|}^{2}+{\left|E_{0y}\right|}^{2}}$ is the field amplitude of **E**_0_(*t*). *β* + Δ*β*(*t*) and *α* + *Δα*(*t*) repress two time-varied polarimetric parameters, the polarization angle and the phase difference between *E*_0_*_x_*(*t*) and *E*_0_*_y_*(*t*), respectively. Here, tan [*β* + Δ*β*(*t*)] = *E*_0_*_y_*(*t*)/*E*_0_*_x_*(*t*), *α* + Δ*α*(*t*) = *α_y_*(*t*) − *α_x_*(*t*), and 0 ≤ *β* + Δ*β*(*t*) ≤ *π*/2, as well as 0 ≤ *α* + Δ*α*(*t*) ≤ 2*π*. *α_x_*(*t*) and *α_y_*(*t*) are the optical phases of *E*_0_*_x_*(*t*) and *E*_0_*_y_*(*t*), respectively. Parameters *β* and *α*, decided by PC_2_, determine the position of the elliptical polarization vector, **E**_0_(*t*), on the Poincaré sphere; while Δ*β*(*t*) and Δ*α*(*t*), decided by PC_1_, are two fast-varied, zero-mean, random parameters, which reflect the SOP evolutions. According to Equation (3), in a practical operation, one can determine the DOP of the output beam by adjusting PC_1_, and select the required SOP by adjusting PC_2_. The overall time-averaging optical intensity of the output beam is I0=Re{E0(t)⋅E0*(t)}/2=(|E0x|2+|E0y|2)/2. Here, the time-average for a partially polarized beam should take enough optical cycles.

### 3.2. Principle of the Balanced Sagnac Sensor

Before analyzing the principle of the balanced Sagnac sensor, it should be emphasized that the output beam from the chaotic fiber ring laser actually is of low coherence. The coherence length, *l_c_*, of the light source is estimated to be less than 1 mm. Therefore, two probe beams meeting at the photodetector will not interfere with each other, if their optical path difference is larger than *l_c_*. This condition can be easily satisfied in this sensor configuration by simply choosing Δ*l* > *l_c_* in a long dual-core fiber cable. With this precondition, the balanced Sagnac sensor can be treated as two individual Sagnac interferometers, which are defined as the upper Sagnac and lower Sagnac interferometers, respectively, as shown in Figure 5c. The output beam of the laser is divided by a 3-dB coupler, OC_2_, into two equal-intensity probe beams (see Figure 5b). Assuming that the coupler is lossless and identical for both polarizations in either direction, the electric fields of two probe beams, defined as **E**_U_(*t*) = (*E_Ux_*(*t*),*E*_U_*_y_*(*t*))^T^ and **E**_L_(*t*) = (*E_Lx_*(*t*), *E_Ly_*(*t*))^T^, are given by:

(4) $$\left(\begin{array}{c} E_{\text{U}}(t)\\ E_{\text{L}}(t) \end{array}\right)=\underset{\left({\text{OC}}_{2}\right)}{\underset{︸}{\frac{1}{\sqrt{2}}\left(\begin{array}{cc} K_{\text{S}} & K_{\text{A}}\\ K_{\text{A}} & K_{\text{S}} \end{array}\right)}}\left(\begin{array}{c} E_{0}(t)\\ 0 \end{array}\right)$$

where **K**_S_ and **K**_A_ are the transfer matrices describing the straight coupling and across coupling of the beam when passing through the coupler, respectively. They are given by:

(5) $$\begin{array}{cc} K_{\text{S}}=\left(\begin{array}{cc} 1 & 0\\ 0 & 1 \end{array}\right), & K_{\text{A}}=\left(\begin{array}{cc} j & 0\\ 0 & j \end{array}\right) \end{array}$$

We assume that the acoustic wave acts on the fiber coil around the point, *D*, which deviates a distance, *r*, from the middle of the loop, as depicted in Figure 5c. A time-dependent phase shift is formed by the acoustic wave between the CW and CCW beams and expressed as Δ*ϕ = ϕ*(*τ*_2_) − *ϕ*(*τ*_1_) for the upper Sagnac interferometer and −Δ*ϕ* = *ϕ*(*τ*_2_) − *ϕ*(*τ*_1_) for the lower Sagnac interferometer. Here, *ϕ*(*t*) is a phase modulation caused by the acoustic wave, *τ*_1_ and *τ*_2_ are two time constants corresponding to propagating time of two counter-propagating beams through the fiber coil from the point, *D*, to the coupler, OC_3_. In addition, there exists another static, nonreciprocal phase shift, *ϕ*_b_, also called the phase bias, which arises from the fiber birefringence. In principle, the birefringence within the fiber coil will create two optical paths for the CW and CCW beams propagating, respectively, with different mode propagating constants [38]. This causes a phase shift between the CW and CCW beams, even without external disturbances. Notice that *ϕ*_b_ has the same sign in two Sagnac interferometers. In the following, we deduce the response function of the balanced Sagnac sensor by using the equivalent optical network theory [39] and simply modeling the birefringent Sagnac loop as a wave-plate with one phase retardation.

In the upper Sagnac interferometer, two output electric fields, $E_{1}^{\text{U}}(t)={\left(E_{1x}^{\text{U}}(t),E_{1y}^{\text{U}}(t)\right)}^{\text{T}}$ and $E_{2}^{\text{U}}(t)={\left(E_{2x}^{\text{U}}(t),E_{2y}^{\text{U}}(t)\right)}^{\text{T}}$, are expressed as:

(6) $$\left(\begin{array}{c} E_{1}^{\text{U}}(t)\\ E_{2}^{\text{U}}(t) \end{array}\right)=\underset{\left({\text{OC}}_{3}\right)}{\underset{︸}{\frac{1}{\sqrt{2}}\left(\begin{array}{cc} K_{\text{S}} & K_{\text{A}}\\ K_{\text{A}} & K_{\text{S}} \end{array}\right)}}\underset{T}{\underset{︸}{\left(\begin{array}{cc} 0 & N\\ N & 0 \end{array}\right)}}\underset{{\text{B}}^{\text{U}}}{\underset{︸}{\left(\begin{array}{cc} B_{CW}^{\text{U}} & 0\\ 0 & B_{\text{CCW}}^{\text{U}} \end{array}\right)}}\underset{\left({\text{OC}}_{3}\right)}{\underset{︸}{\frac{1}{\sqrt{2}}\left(\begin{array}{cc} K_{\text{S}} & K_{\text{A}}\\ K_{\text{A}} & K_{\text{S}} \end{array}\right)}}\left(\begin{array}{c} E_{\text{U}}(t)\\ 0 \end{array}\right)$$

and in the lower Sagnac interferometer, two output electric fields, $E_{1}^{\text{L}}(t)={\left(E_{1x}^{\text{L}}(t),E_{1y}^{\text{L}}(t)\right)}^{\text{T}}$ and $E_{2}^{\text{L}}(t)={\left(E_{2x}^{\text{L}}(t),E_{2y}^{\text{L}}(t)\right)}^{\text{T}}$, are expressed as:

(7) $$\left(\begin{array}{c} E_{1}^{\text{L}}(t)\\ E_{2}^{\text{L}}(t) \end{array}\right)=\underset{\left({\text{OC}}_{3}\right)}{\underset{︸}{\frac{1}{\sqrt{2}}\left(\begin{array}{cc} K_{\text{S}} & K_{\text{A}}\\ K_{\text{A}} & K_{\text{S}} \end{array}\right)}}\underset{T}{\underset{︸}{\left(\begin{array}{cc} 0 & N\\ N & 0 \end{array}\right)}}\underset{{\text{B}}^{\text{L}}}{\underset{︸}{\left(\begin{array}{cc} B_{CW}^{\text{L}} & 0\\ 0 & B_{\text{CCW}}^{\text{L}} \end{array}\right)}}\underset{\left({\text{OC}}_{3}\right)}{\underset{︸}{\frac{1}{\sqrt{2}}\left(\begin{array}{cc} K_{\text{S}} & K_{\text{A}}\\ K_{\text{A}} & K_{\text{S}} \end{array}\right)}}\left(\begin{array}{c} 0\\ E_{\text{L}}(t) \end{array}\right)$$

where **T** denotes a transposition matrix, which is used to exchange the port number of OC_3_ when beams go back to the coupler and **N** is a 2 × 2 matrix used to change the sign of the *x*-component of the electric field of the polarization beam after propagating through the fiber coil [40]. It is necessary when one employs a polarization model to analyze the fiber Sagnac interferometer. This matrix is defined as:

(8) $$N=\left(\begin{array}{cc} -1 & 0\\ 0 & 1 \end{array}\right)$$

**B**^U^ and **B**^L^ are the transfer matrix of the upper Sagnac interferometer and the lower Sagnac interferometer, respectively. **B**^U^ contains two Jones matrices, $B_{CW}^{\text{U}}$ and $B_{\text{CCW}}^{\text{U}}$. **B**^L^ contains another two Jones matrices, $B_{CW}^{\text{L}}$ and $B_{\text{CCW}}^{\text{L}}$. They represent the phase shifts two probe beams experienced; when beams travel in the fiber coil in the CW and CCW direction, in two Sagnac interferometers, respectively. They are given by:

(9) $$\begin{array}{cc} B_{CW}^{\text{U}}=\left(\begin{array}{cc} e^{j(\phi_{\text{b}}+\Delta \phi )/2} & 0\\ 0 & e^{j(\phi_{\text{b}}+\Delta \phi )/2} \end{array}\right), & B_{\text{CCW}}^{\text{U}}=\left(\begin{array}{cc} e^{-j(\phi_{\text{b}}+\Delta \phi )/2} & 0\\ 0 & e^{-j(\phi_{\text{b}}+\Delta \phi )/2} \end{array}\right) \end{array}$$

for the upper Sagnac interferometer and:

(10) $$\begin{array}{cc} B_{CW}^{\text{U}}=\left(\begin{array}{cc} e^{j(\phi_{\text{b}}-\Delta \phi )/2} & 0\\ 0 & e^{j(\phi_{\text{b}}-\Delta \phi )/2} \end{array}\right), & B_{\text{CCW}}^{\text{U}}=\left(\begin{array}{cc} e^{-j(\phi_{\text{b}}-\Delta \phi )/2} & 0\\ 0 & e^{-j(\phi_{\text{b}}-\Delta \phi )/2} \end{array}\right) \end{array}$$

for the lower Sagnac interferometer.

Four time-averaged interference signals obtained, respectively, with Equations (6) and (7) are presented as follows:

(11) I1U=12Re{E1U(t)⋅E1U*(t)}=I04{1+cos(Δϕ+ϕb)}I2U=12Re{E2U(t)⋅E2U*(t)}=I04{1−cos(Δϕ+ϕb)}I1L=12Re{E1L(t)⋅E1L*(t)}=I04{1−cos(Δϕ−ϕb)}I2L=12Re{E2L(t)⋅E2L*(t)}=I04{1+cos(Δϕ−ϕb)}

Finally, the output of the balanced Sagnac sensor is obtained through a differential operation with two sets of interference signals, $I_{1}=I_{1}^{\text{U}}+I_{1}^{\text{L}}$ and $I_{2}=I_{2}^{\text{U}}+I_{2}^{\text{L}}$, obtained above, and expressed as:

(12) $$\begin{array}{ll} I_{\text{out}} & =I_{2}-I_{1}\\ & =\frac{I_{0}}{2}(1+\sin\phi_{\text{b}}\sin\Delta \phi )-\frac{I_{0}}{2}(1-\sin\phi_{\text{b}}\sin\Delta \phi )\\ & =I_{0}\sin\phi_{\text{b}}\sin\Delta \phi \\ & =I_{0}V\sin\Delta \phi \end{array}$$

where *V* = sin *ϕ*_b_ is defined as the fringe visibility of the sensor output or the scale factor. When *ϕ*_b_ = *π*/2, yielding *V* = 1, the sensor output will achieve its maximum visibility and higher SNR. Equation (12) represents a response function of the balanced Sagnac sensor. It should be noted that this balanced Sagnac sensor has an inherent property of operating at the quadrature point of its response curve, and any changes in the phase bias, *ϕ*_b_, only affect the visibility, not the working point. This is distinct from other ordinary types of fiber Sagnac interferometers, where only a single probe beam is employed. It also should be noted that since the PC_2_ can statistically determine the central SOP of the laser beam, in practical operations, it is possible to change the fringe visibility by adjusting PC_2_. For this, detailed explanations will be given in Section 5.

## 4. Experimental Results

A prototype of the sensor system based on the proposed configuration was built. In this prototype, the chaotic fiber ring laser consisted of a C-band EDFA, a 1 × 2, 30:70 fiber coupler (OC_1_) and a 1 × 2, 3-dB fiber coupler (OC_2_), as well as two fiber squeeze-type PCs (PC_1_ and PC_2_). The total length of the fiber ring was estimated to be about 14 m, including a 9 m-long active fiber in the EDFA and other passive fibers contained in the PC_1_ and OC_1_, as well as in the EDFA. The average output power of the laser was preset at around 0 dBm by using an optical attenuator (ATT). In the balanced Sagnac sensor, two three-port, fiber-optic circulators (CIR.) were used. The sensor head was the same as one used in the previous experiment, which included a 1 km-long fiber coil and a 2 × 2, 3-dB fiber coupler (OC_3_). The sensor head was connected to the sensor system with a 10 m-long, dual-core fiber cable. Two identical, 1.25-GHz photodiodes combined with two electric front-end receivers with the same gain and bandwidth (125 MHz) were employed. Two detected current signals after passing through the respective high-pass filter with a cut-off frequency of 10 kHz, were fed into a digital storage oscilloscope for data processing (subtracting) and waveform analyses or fed into an optical spectrum analyzer for spectrum observations. Based on this prototype, a series of experiments were carried out for investigating the performances of the sensor system, as well as for evaluating the feasibility of the proposed sensor technology. In each subsection below, we will present the experimental results on the characterization test of the laser, the performance evaluations of the balanced Sagnac sensor, as well as the actual detections of PD signals generated in our laboratory environment.

### 4.1. Characterization Test of the Chaotic Laser Source

**Figure 6.**
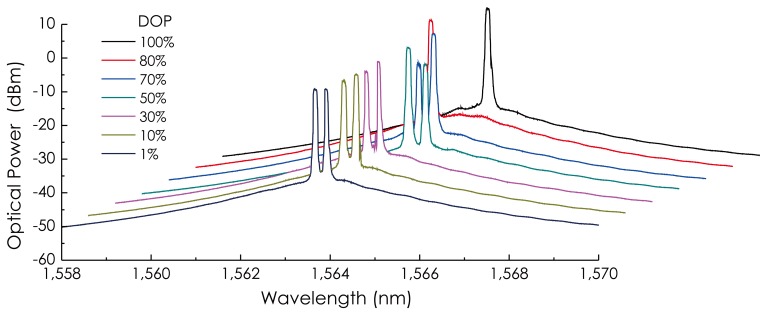
Output spectra of the chaotic fiber ring laser at different DOP values.

The output beam of the laser was observed at the point, m, in the schematic of the sensor configuration (see Figure 4). Figure 6 is a group of the output spectrum of the laser at different DOP values set by adjusting PC_1_. From these results, it could be observed that the laser oscillated in a stable, dual-wavelength mode as DOP < 80%, in an unstable, single-wavelength mode as 80% < DOP < 98%, as well as in a stable, single-wavelength mode as DOP ≈ 100%. The oscillating wavelength of the laser changed within 1,563.63–1,563.98 nm. This dual-wavelength oscillating arises from the birefringence of the fiber, which creates two optical cavities with different lengths within the fiber ring.

**Figure 7.**
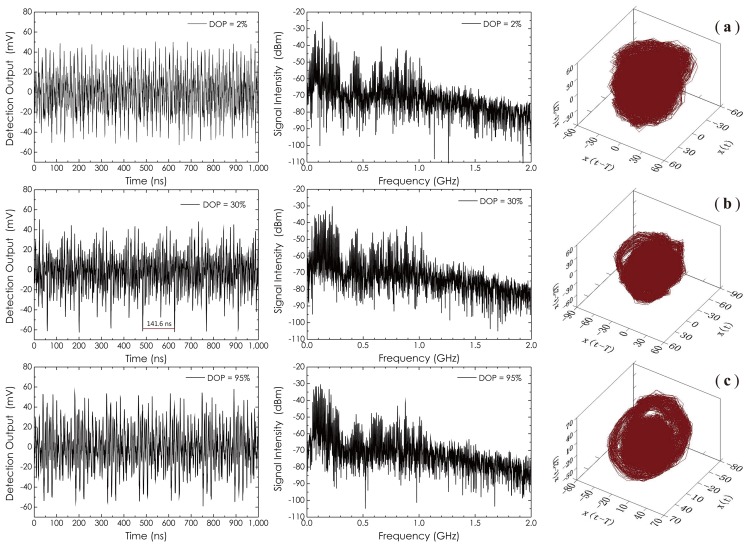
Time waveforms, frequency spectra of the output beam at different DOP values, as well as corresponding phase space trajectories with a delay *T* = 7, where: (**a**) DOP = 2%; (**b**) DOP = 30%; and (**c**) DOP = 95%.

Figure 7 has three sets of time waveforms, frequency spectra and corresponding phase space trajectories of the output beam at different DOP values. The phase-space graphs usually are used in nonlinear physics to reveal the attractors and to calculate the embedded dimension in the time series from a dynamical system (DS) for the reconstruction of the DS [41]. The parameter, *T*, is a time delay, which is usually chosen experientially; here, *T* = 7 was selected. The scroll chaotic attractor in each phase-space graph (DOP = 30% and DOP = 95%) can be clearly identified. However, in Figure 7a (DOP = 2%), where the white noises become dominant, the identification of the attractor becomes difficult. From the measured time waveforms, one also can confirm that the output beam has a characteristic of chaotic intensity fluctuations, especially as DOP = 30%, where an obvious frame structure, called the periodic evolutions of the system state, can be observed. The double frame period (2*τ*_R_) measured in this waveform is about 141.6 ns, which corresponds to a ring length of 14.2 m. The frequency distribution in each chaotic waveform is over 1 GHz, which almost is an upper limitation (≈ 1.25 GHz) of the frequency response of the detector used in this measurement.

**Figure 8.**
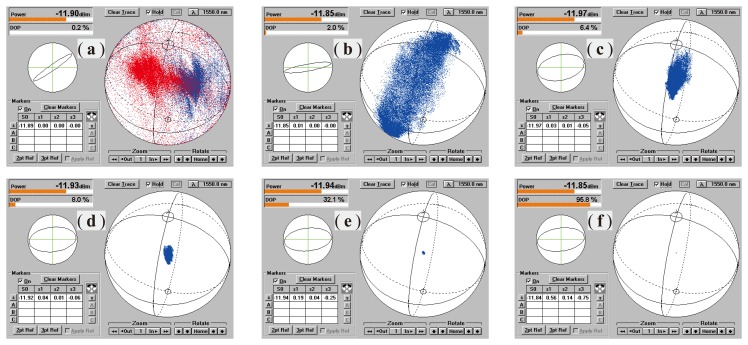
Distributions of the SOP of the output beam on the Poincaré sphere measured at different DOP values, changing from 0.2% (**a**) to 95.8% (**f**).

Figure 8 shows a group of SOP distributions of the output beam on the Poincaré sphere measured at different DOP values, changed from 0.2% to 95.8% by adjusting PC_1_. It is obvious that with the DOP decreasing, the spreading area or fluctuation degree of the SOP will increase, while its center, however, basically keeps constant. This center only can be altered by adjusting PC_2_.

**Figure 9.**
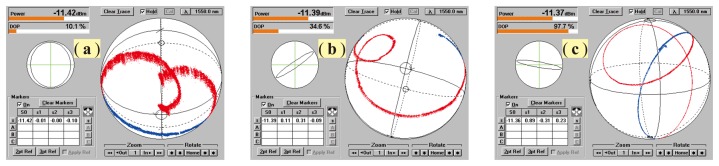
Measured varying traces of SOP with a fixed DOP as PC_2_ was continuously adjusted, where DOP ≈ 10% (**a**), DOP ≈ 35% (**b**) and DOP ≈ 98% (**c**), respectively.

**Figure 10.**
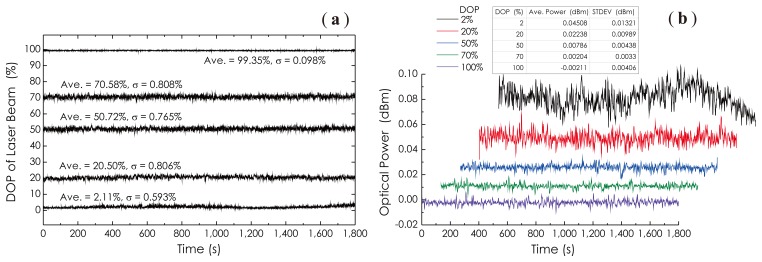
Measured results on the long-term stabilities of: (**a**) DOP; and (**b**) the optical power of the laser beam at different DOP values.

From these results, one can observe that the averaged output power (≈ −11.91 dBm) of this chaotic fiber ring laser is independent of the DOP; even the DOP is adjusted with a large span. It is a very favorable and important feature, especially in the field of optical measurements, where often, a power-stable light source is required. This feature comes from the very low polarization dependent gain of the EDFA, as well as the minimal polarization dependent losses (PDL) of all of the fiber-optic components used in this prototype. Figure 9 shows three measured SOP traces at different DOPP values as PC_2_ was continuously adjusted where the DOP was kept constant. These results reveal that the SOP of the laser beam can be completely controlled with respect to its distribution and location on the Poincaré sphere by adjusting PC_1_ and PC_2_, respectively. The long-term stability of the DOP, as well as the long-term stability of the output power of the laser at different DOP values were investigated experimentally, and the results are demonstrated in Figure 10, respectively. During a measurement period of 1,800 s, the maximum variation of the DOP is less than 1% as DOP ≈ 20.5%, and the maximum power fluctuation in the whole DOP range is less than 0.05 dBm. However, it is obvious that with the DOP decreasing, power fluctuations increased rapidly. It was guessed that in the low DOP state, the random intensity noises in this chaotic fiber ring laser became dominant.

### 4.2. Performance Evaluation of the Balanced Sagnac Sensor

**Figure 11.**
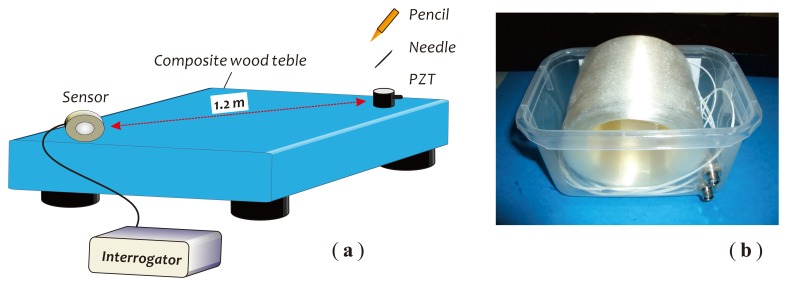
Schematic of the test platform (**a**) used for sensor performance evaluations; (**b**) a photo of the fiber sensor head used in the experiments. PZT, piezoelectric transducer.

**Figure 12.**
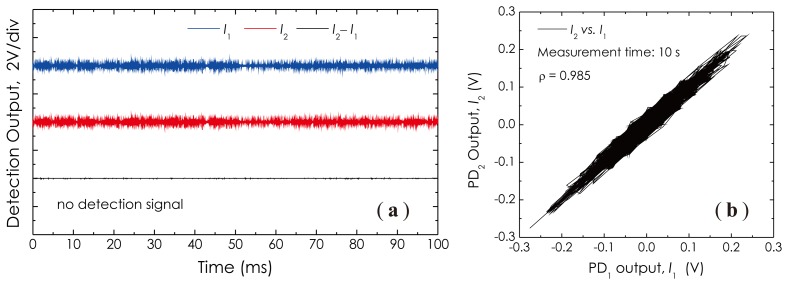
Measured results: (**a**) three signal waveforms from the sensor; (**b**) cross-correlation of two detection outputs.

The sensor performances with respect to the sensitivity and frequency response were investigated by utilizing a composite wood table as the test platform, as depicted in Figure 11a. Figure 11b is a photo of the fiber sensor head made with a 1 km-long single-mode fiber. The sensor head was put on the table near a corner where the silicon grease was painted for getting a good acoustic coupling. Another corner across the table with a distance of ∼1.2 m toward the sensor head was used as the test place where a 2 cm-long, small metal needle, a HB pencil and a PZT actuator were employed as tools to generate the required acoustic wave signals for the sensor performance testing. The optical power of the laser beam was set at about 0 dBm through an optical attenuator. At first, in Figure 12a, we demonstrate three detected signal waveforms obtained in the absence of external acoustic disturbances. Figure 12b is a measured result related to the cross-correlation property of two detection outputs, *I*_1_ and *I*_2_, obtained during a measurement period of 10 s. This correlation graph represents the similarity or cross-correlation degree of two outputs of the balanced Sagnac sensor. The cross-correlation coefficient, *ρ*, is calculated with a formula [31]:

(13) $$\rho =\frac{\langle \left[X(t)-\langle X(t)\rangle ][Y(t)-\langle Y(t)\rangle \right]\rangle }{\sqrt{\langle {\left|X(t)-\langle X(t)\rangle \right|}^{2}\rangle \langle {\left|Y(t)-\langle Y(t)\rangle \right|}^{2}\rangle }}$$

where *X*(*t*) and *Y*(*t*) denote two detection signals, *I*_1_ and *I*_2_, respectively. The change range of *ρ* is − 1 ≤ *ρ* ≤ 1. A larger |*ρ*| value indicates the better balance property of the sensor. Utilizing the data collected in Figure 12b, we calculated the cross-correlation coefficient, having *ρ* ≈ 0.985. From these results, it is obvious that two outputs of the balanced Sagnac sensor are well balanced in the signal intensity, as well as in the phase. There are many obvious intensity fluctuations appearing in both *I*_1_ and *I*_2_, which are the common-mode intensity noises; however, in the final output, *I*_2_ − *I*_1_, these noises are reduced greatly, which indicates a substantial enhancement of the SNR of the sensor system with this balanced detection configuration.

**Figure 13.**
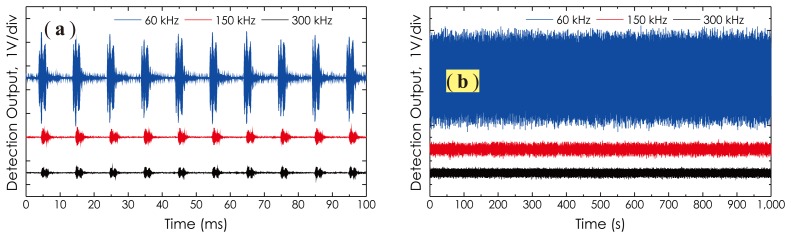
Measured signal waveforms used for investigating: (**a**) frequency response; (**b**) long-term stability.

The frequency response of the sensor prototype in the ultrasonic frequency range was investigated by employing a PZT actuator (MISTRAS, model: **R**15*α*) to generate three AE signals at the ultrasonic frequency of 60 kHz, 150 kHz and 300 kHz, respectively. The PZT actuator was driven by a portable AE signal generator (FieldCAL AE Signal Generator, MISTRAS Group Inc. Princeton Junction, NJ, USA), working in burst mode with a 10-ms repetition period and a 90-dB amplitude. The detected AE signal waveforms from *I*_2_ − *I*_1_ are illustrated in Figure 13a. Figure 13b is a collection of the signal waveforms measured at each ultrasonic frequency during a measurement period of up to 1,000 s. In this experiment, medium-amplitude, randomly-varied vibration from a speaker also was applied to the fiber coil. The DOP of the laser beam in this case was preset at 10%, which is sufficient to cover most birefringence fluctuations induced by external vibrations. From these results, it is clear that this sensor prototype worked well with respect to the detection of high-frequency AE signals from 60 kHz to 300 kHz, and its long-term stability also was excellent, although there existed external vibrations disturbing the fiber coil. Moreover, due to the DOP tunable function of the light source, the amplitude fluctuations in the detection signals are not obvious. It should be noted that although the sensor head was designed with an optimal detection sensitivity at about 100 kHz, due to the large acoustic absorption the table material possesses for high-frequency ultrasonic waves, the actual signal amplitudes detected in the high frequency range (150 kHz, as well as 300 kHz) were obviously abated.

The calculation of the optimal frequency response of the sensor head is made based on the consideration of getting a maximum net phase shift when an acoustic wave disturbs the fiber coil. This corresponds to getting a maximum time difference, Δ*t*, between the CW and CCW beams. With this consideration, we have Δ*t* = *nL*/*c*, where *L* is the fiber length, *n* is the refractive index of the fiber core and c is the speed of light in vacuum. A roll-off response in the fiber coil occurs at *f_s_* = 1/(2Δ*t*) [42], at which there is a maximum net phase shift. Thus, we can calculate the optimal frequency response of the sensor head with *f_s_* = *c*/(2*nL*). When *L* = 1 km and *n* ≈ 1.48, we obtain *f_s_* ≈ 100 kHz.

**Figure 14.**
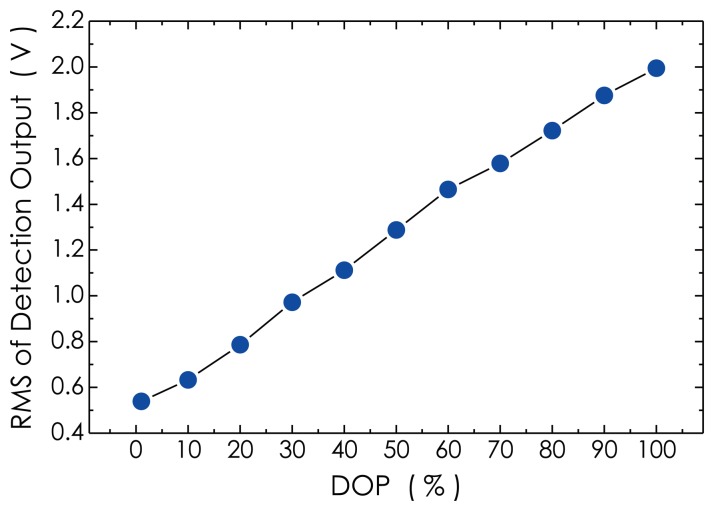
Measured result on the relation between sensor sensitivity and the DOP of the laser beam.

The measurement result on the RMS of the detection outputs *versus* the DOP of the laser beam is presented in Figure 14, which represents a relation between the sensor sensitivity and the DOP value used. In this experiment, a 60-kHz, burst-mode ultrasonic wave signal was detected in the absence of external vibrations, and the RMS value of amplitude of the detected signal at each DOP was calculated. From the result, it can be seen that with the DOP reducing from 100% down to 1%, the RMS magnitude linearly drops from its maximum value of about 1.995 V down to 0.539 V. This means that the sensitivity of the sensor system will obviously be reduced as the DOP drops. At present, the Lyot-type depolarizers have been widely employed in most optical measurement systems to effectively eliminate the polarization dependency. However, the DOP value of the light source equipped with this kind of depolarizer is fixed, generally, lying between 5% and 10%, which means that the sensitivity of the optical measurement system is relatively low, compared with using a high-DOP light source. According to our experimental investigations referred to in Section 2, the polarization fading is variable, depending on the actual operational environment. Therefore, the use of the DOP-tunable light source in a sensor system is reasonable with respect to effectively suppressing the polarization fading, as well as enhancing the sensitivity, compared with using a DOP-fixed light source.

The setting of a suitable DOP basically depends on the maximum fading span. In principle, the changes in the optical intensity of the signal beam, induced by the fluctuations of the SOP of the laser beam, should be able to cover the overall fading induced by external disturbances, as schematically described in Figure 2. Too low and too high of a DOP adopted in actual applications will easily result in the excessive decrease of the average detection level and the insufficient suppression of the polarization fading, respectively. Owing to the limitation of the bandwidth of the detection system employed in our experiments, at the present stage, however, convincing experimental data for explaining this concept relating to a high-speed optical intensity variation (up to several gigahertz) arising in the signal beam, produced by the fluctuations of the SOP of the laser beam have not been obtained yet.

**Figure 15.**
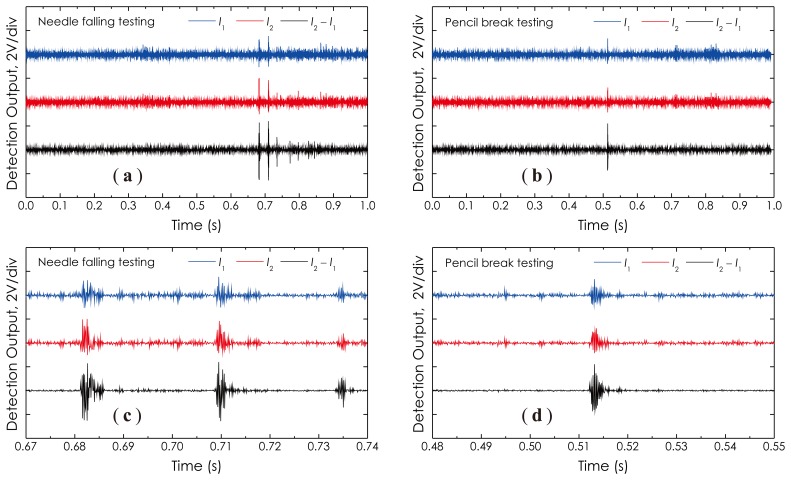
Measured waveforms from: (**a**) needle falling; and (**b**) pencil lead breaking. (**c**,**d**) Two corresponding enlarged views.

Next, we demonstrate several signal waveforms detected under the following conditions or actions: (1) the needle free-falling from a 2-cm height down to the wood table; and (2) the pencil lead breaking, in order to generate the AE signal in a unique pattern. The detected results are presented in Figure 15a,b, respectively. Figure 15c,d are two corresponding enlarged views around detection pulses, as illustrated in Figure 15a,b, respectively. With these waveforms, one can determine the different AE signal patterns the sensor actually responded to under different exciting conditions. For example, in Figure 15a, multiple groups of the pulse can be seen, which indicates that, actually, there existed a springing process after the metal needle free-dropped down to the wood table, while in Figure 15b, only one pulse could be seen, which represents that just one AE occurred as the pencil lead was broken. It should be noted here that the SNR of the final detection signal, *I*_2_ − *I*_1_, as the lowest trace shown in each graph, is obviously higher than that of any other detection signal, *I*_1_ or *I*_2_. Theoretically a 3-dB enhancement in the SNR of the detection signal can be obtained if two receivers are completely identical in terms of the detection performance. Furthermore, it is observed that each signal waveform is almost symmetrical in amplitude around the base line (zero volt), which indicates that this sensor actually operated at its quadrature point, as predicted by Equation (12).

### 4.3. Discharge Detections

**Figure 16.**
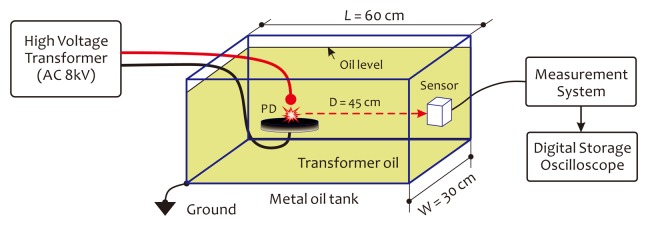
Experimental setup for discharge detections.

With our sensor prototype, in our laboratory environment, we detected the AE signals produced through HV discharges occurring within a metal tank, with and without filling, with the transformer oil (mineral oil), respectively. This work was to verify the feasibility of the proposed sensor technology, as well as to investigate the sensitivity of the sensor prototype for sensing the discharge-induced AE signals. The experimental setup for discharge detections is schematically illustrated in Figure 16, in which a pair of pin-plate-type electrode sets was used to generate different types of discharge for observations. The sensor head was attached to the shell of the tank, facing the electrodes, fixed by several magnet blocks. The distance between the sensor head and the HV electrodes was about 45 cm. The gap of two electrodes was fixed at about 8 mm, while the imposed AC high voltage was adjustable from 0 V to 8 kV. The AC high voltage was generated by an HV transformer with a variable AC low-voltage input. In this experiment, the room environment was controlled at 25 °C and 65% RH (Relative Humidity). In order to achieve high sensing sensitivity, the DOP of the laser beam was preset at ∼95%.

**Figure 17.**
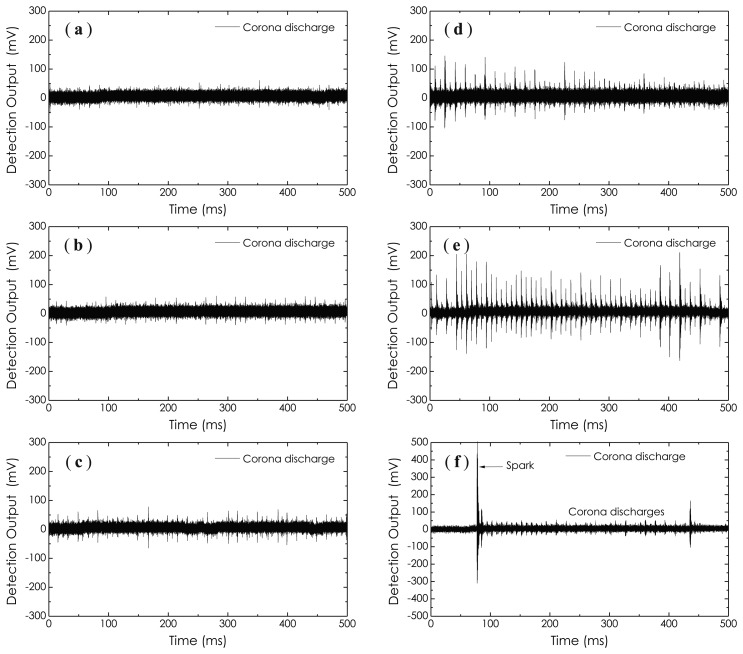
Experimental results on the sensing of corona discharges occurring within an empty metal tank. From (**a**) to (**e**), the imposed AC high voltage increased gradually, large spark pulses (**f**).

Figure 17 illustrates a group of experimental results on the detections of corona discharges occurring within an empty metal tank. In this experiment, when the AC high voltage imposed on the electrodes reached about 2 kV, the corona discharges (small and periodic pulses) began to appear and enhanced gradually as the AC high voltage increased. This process can be illustrated graphically by the waveforms presented in Figure 17a–e. Sometimes, large spark pulses emerged suddenly; see Figure 17f. It should be noted that the occurrence of the corona discharge is not only directly related to the voltage imposed on the electrodes, but also is a function of relative humidity in the room. As relative humidity in the room increases, the occurrence probability, as well as the intensity of the corona discharge will significantly increase.

**Figure 18.**
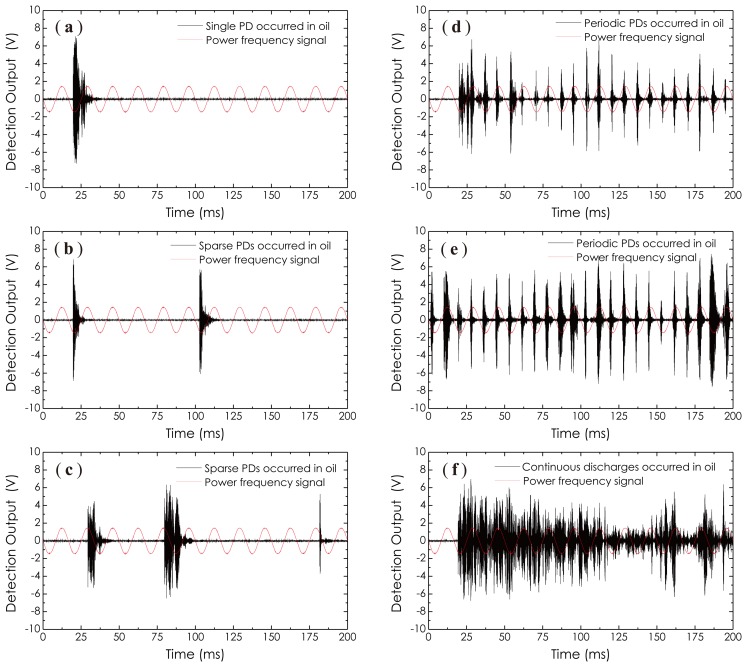
Experimental results on the sensing of partial discharges occurring within a metal tank filling with transformer oil.

When the metal tank was full of transformer oil, the corona discharges disappeared. However, when the AC high voltage continuously increased and exceeded 6.5 kV, partial discharges arose. The results on the detections of partial discharges are shown in Figure 18, where a power frequency signal is introduced as a phase reference. It should be emphasized here that in an AC supply case, all discharges, including corona discharges, as well as partial discharges, are phase-relevant to the AC power frequency. The one seen in Figure 18a is a single PD pulse appearing when the AC high voltage for the first time arrived at 6.5 kV. After that, the AC high voltage was maintained at this level for a while. We observed that the numbers of PD pulse increased as time went on, but, still, in the form of sparse pulses. These results are presented in Figure 18b,c. When the AC high voltage further increased and exceeded 7.2 kV, periodic partial discharges as a PD pulse train arose and are shown in Figure 18d,e. From these waveforms, one can see that the average intensity of the PD pulse train enhanced as the the imposed AC high voltage increased. Here, the AC high voltage used in Figure 18e was higher than that used in Figure 18d; therefore, the average amplitude of PD pulses shown in Figure 18e is higher than that shown in Figure 18d. As the amount of discharge activity increased, the transformer oil became dirty, and the oil temperature also increased rapidly Finally, severe discharges happen, accompanied by smoke and light. Figure 18f recorded these discharge-induced AE signals, in which the previous periodic PD pulses became a group of continuous clutter waveforms. All experimental results presented in Figures 17 and 18 demonstrate the feasibility of the proposed sensor technology well, which can be well used in the power industry field in the future with superior sensitivity for the PD monitoring of power transformers.

## 5. Discussions

Depending on Equation (12), it is clear that the proposed balanced Sagnac sensor can inherently operate at its quadrature point with the highest sensitivity for sensing very weak acoustic wave signals, such as the PD signals. The fringe visibility is a function of the phase bias, *ϕ*_b_, so it may vary with the operational environment, in turn causing the signal fading, however, which can be well suppressed by setting a suitable DOP through adjusting PC_1_. In addition, as mentioned before, since the central SOP of the fiber ring laser beam can be statistically determined through adjusting PC_2_, in practical operations, it is possible to tune the fringe visibility by selecting a suitable statistical SOP of the laser beam through adjusting PC_2_. The reason for doing this can be explained as follows.

According to the previous research in [16,20,43], the fringe visibility generally is dependent on the state of polarization of the input light and the fiber birefringence, although it is impossible to give a well defined explanation with our polarization analytical model, due to its simplicity without considering the polarization mode coupling in the fiber. In a more general polarization analytical model adopted in some literature, the linear birefringence and circular birefringence of the fiber, as well as the polarization mode coupling have been considered [20,23,44]. In this kind of model, the SOP of the input light, in terms of the polarization angle, *β*, as well as the phase difference, *α*, will be included, as critical parameters, into the response function of the sensor to affect the sensor performances. Therefore, it is possible to tune the fringe visibility through changing the SOP of the input light. However, owing to the complexity of this kind of analytical model, it is difficult to get clear analytical expressions to clarify this behavior of the interferometer, and many results can only be obtained through numerical calculations.

In our previous experiments, the DOP tunable function of the laser source for suppressing the polarization fading noises has not been widely investigated, yet, owing to the limitation of the experimental conditions in our laboratory. This work will be continued in the future in actual industrial environments, such as in a power transformer, in which much more definite conditions will be utilized and more credible experimental results can be obtained.

## 6. Conclusions

In this article, we presented a novel fiber-optic acoustic sensor using a balanced Sagnac sensor and an EDFA-based fiber ring laser with the DOP tunable function. The basic principles of the sensor system were introduced. As a technical background, we carried out an experiment for investigating the intensity fluctuations in detection signals, which were caused by the time-varying birefringence in the fiber coil, resulting from external vibrations. The DOP-tunable fiber ring laser proposed in this configuration actually operates in chaotic mode and is capable of providing abundant polarization dynamics to output a DOP-controlled, partially-polarized laser beam. This DOP-tunable laser can be used in the fiber optic sensing field for effectively suppressing the polarization fading noises appearing in any interference-type sensor system. Some experiments for evaluating the laser source and the balanced Sagnac sensor were carried, and the results have been demonstrated. For this DOP-tunable laser, the DOP of the laser beam could be adjusted continuously from 0.2% to ∼100% without obvious power fluctuations (<0.05 dBm). The balanced Sagnac sensor could detect acoustic wave signals produced through different actions with the high SNR, such as a needle falling and pencil lead breaking. The designed sensor head had a better frequency response property, especially in detecting high-frequency ultrasonic wave signals up to 300 kHz. The experiments on the detections of AE signals produced through HV discharges were carried out in our laboratory. The experimental results showed that the proposed fiber optic acoustic sensor could detect AE signals produced by corona discharges, as well as partial discharges occurring inside a metal tank, well, with and without the transformer oil, respectively.

## References

[1] Boggs S.A. (1990). Partial discharge: Overview and signal generation. IEEE Electr. Insul. Mag..

[2] Udd E., Udd E. (1991). The emergence of fiber optic sensor technology. Fiber Optic Sensors : An Introduction for Engineers and Scientists.

[3] Culshaw B. (2000). Fiber optics in sensing and measurement. IEEE J. Sel. Topics Quantum Electr..

[4] Bosselmann T. Innovative applications of fibre-optic sensors in energy and transportation.

[5] Posada J.E., Rubio-Serrano J., Garcia-Souto J.A. All-fiber interferometric sensor of 150 kHz acoustic emission for the detection of partial discharges within power transformers.

[6] Zargari A., Blackburn T.R. Modified optical fibre sensor for PD detection in high-voltage power equipment.

[7] Dong B., Han M., Sun L., Wang J., Wang Y., Wang A. (2008). Sulfur hexafluoride-filled extrinsic Fabry-Pérot interferometric fiber-optic sensors for partial discharge detection in transformers. IEEE Photon. Technol. Lett..

[8] Zhao Z., MacAlpine M., Demokan M.S. (2000). The directionality of an optical fiber high-frequency acoustic sensor for partial discharge detection and location. J. Light. Technol..

[9] Blackburn T.R., Phung B.T., James R.E. Optical fibre sensor for partial discharge detection and location in high-voltage power transformer.

[10] Song L., Cooper K.L., Wang Z., Wang A., Liu Y. (2006). Position location of partial discharges in power transformers using fiber acoustic sensor arrays. Opt. Eng..

[11] Chin K.K., Sun Y., Feng G., Georgiou G.E., Guo K., Niver E., Roman H., Noe K. (2007). Fabry-Perot diaphragm fiber-optic sensor. Appl. Opt..

[12] Dong B., Han M., Wang A. (2012). Two-wavelength quadrature multipoint detection of partial discharge in power transformers using fiber Fabry-Perot acoustic sensors. Fiber Opt. Sens. Appl. IX Proc. SPIE.

[13] Perez I., Cui H.L., Udd E. (2001). Acoustic emission detection using fiber Bragg gratings. Smart Struct. Mater..

[14] Rosenthal A., Razansky D., Ntziachristos V. (2011). High-sensitivity compact ultrasonic detector based on a pi-phase-shifted fiber Bragg grating. Opt. Lett..

[15] Udd E. (1983). Fiber-optic acoustic sensor based on the Sagnac interferometer. Single Mode Opt. Fibers Proc. SPIE.

[16] Jang T.S., Lee S.S., Kwon I.B., Lee W.J., Lee J.J. (2002). Noncontact detection of ultrasonic waves using fiber optic Sagnac interferometer. IEEE Trans. Ultrason. Ferroelect. Freq. Contr..

[17] Fomitchov P.A., Krishnaswamy S., Achenbach J.D. (2000). Extrinsic and intrinsic fiber optic Sagnac ultrasound sensors. Opt. Eng..

[18] Guan B.O., Tan Y.N., Tam H.Y. (2009). Dual polarization fiber grating laser hydrophone. Opt. Express.

[19] Carolan T.A., Reuben R.L., Barton J.S., Jones J.D.C. (1997). Fiber-optic Sagnac interferometer for noncontact structural monitoring in power plant applications. Appl. Opt..

[20] Pavlath G.A., Shaw H.J. (1982). Birefringence and polarization effects in fiber gyroscopes. Appl. Opt..

[21] Vakoc B.J., Digonnet M.J.F., Kino G.S. (2003). Demonstration of a folded Sagnac sensor array immune to polarization-induced signal fading. Appl. Opt..

[22] Hotate K., Samukawa S. (1990). Drift reduction in an optical heterodyne fiber gyro. Appl. Opt..

[23] Rochford K.B., Day G.W., Forman P.R. (1994). Polarization dependence of response functions in 3 × 3 Sagnac optical fiber current sensors. J. Light. Technol..

[24] Rashleigh S.C. (1983). Origins and control of polarization effects in single-mode fibers. J. Light. Technol..

[25] Burns W.K., Moeller R.P., Villarruel C.A., Abebe M. (1984). All-fiber gyroscope with polarization-holding fiber. Opt. Lett..

[26] Burns W.K., Kersey A.D. (1992). Fiber-optic gyroscopes with depolarized light. J. Light. Technol..

[27] Burns W.K. (1983). Degree of polarization in the Lyot depolarizer. J. Light. Technol..

[28] Wang J.S., Costelloe J.R., Stolen R.H. (1999). Reduction of the degree of polarization of a laser diode with a fiber Lyot depolarizer. IEEE Photon. Technol. Lett..

[29] Wiggeren G.D.V., Roy R. (1999). High-speed fiber-optic polarization analyzer: Measurements of the polarization dynamics of an erbium-doped fiber ring laser. Opt. Commun..

[30] Wiggeren G.D.V., Roy R. (2002). Communication with dynamically fluctuating states of light polarization. Phys. Rev. Lett..

[31] Wang L., Wu W., Fang N., Huang Z. Experimental study on chaotic optical communication with PolSK modulation technology.

[32] Falquier D.G., Digonnet M.J.F., Shaw H.J. (2001). A depolarized Er-doped superfluorescent fiber source with improved long-term polarization stability. IEEE Photon. Technol. Lett..

[33] Williams Q.L., García-Ojalvo J., Roy R. (1997). Fast intracavity polarization dynamics of an erbium-doped fiber ring laser: Inclusion of stochastic effects. Phys. Rev. A.

[34] Spammer S.J., Swart P.L. (1993). A quadrature phase tracker for open-loop fiber-optic gyroscopes. IEEE Trans. Circuits Syst.–I: Fundam. Theory Appl..

[35] Lin H., Lin W.W., Chen M.H. (1999). Modified in-line Sagnac interferometer with passive demodulation technique for environmental immunity of a fiber-optic current sensor. Appl. Opt..

[36] Abarbanel H.D.I., Kennel M.B., Buhl M., Lewis C.T. (1999). Chaotic dynamics in erbium-doped fiber ring lasers. Phys. Rev. A..

[37] Damask J.N. (2005). Vectorial Propagation of Light. Polarization Optics in Telecommunications.

[38] Ulrich R., Johnson M. (1979). Fiber-ring interferometer: Polarization analysis. Opt. Lett..

[39] Kolkiran A., Agarwal G.S. (2007). Heisenberg limited Sagnac interferometry. Opt. Express.

[40] Mortimore D.B. (1988). Fiber loop reflectors. J. Light. Technol..

[41] Anishchenko V.S., Astakhov V., Vadivasova T., Neiman A., Schimansky-Geier L. (2007). Reconstruction of Dynamical Systems. Nonlinear Dynamics of Chaotic and Stochastic Systems: Tutorial and Modern Developments.

[42] Harvey D., McBride R., Barton J.S., Jones J.D.C. (1992). A velocimeter based on the fiber optic Sagnac interferometer. Meas. Sci. Technol..

[43] Chtcherbakov A.A., Swart P.L. (1998). Polarization effects in the Sagnac-Michelson distributed disturbance location sensor. J. Light. Technol..

[44] Burns W.K., Chen C.L, Moeller R.P. (1983). Fiber-optic gyroscopes with broad-band sources. J. Light. Technol..

